# A one-minute resolution global horizontal irradiance dataset recorded in Malaysia for photovoltaic system modelling and validation

**DOI:** 10.1016/j.dib.2026.113242

**Published:** 2026-09-04

**Authors:** Chun Lim Siow, Chee Wern Hew, Ousama M.T. Ajami, Rodney H.G. Tan

**Affiliations:** a Centre for Electric Energy and High Voltage Engineering, COE Robotics & Sensing Technologies Multimedia University, Cyberjaya, Malaysia; b Faculty of Engineering, Technology & Built Environment, UCSI University, No.1, Jalan Menara Gading, UCSI Heights, 56000 Cheras, Kuala Lumpur, Malaysia

**Keywords:** Solar irradiance, Global horizontal irradiance, Solar Insolation, Photovoltaic system

## Abstract

High resolution solar irradiance data are essential for the reliable modelling and validation of solar photovoltaic (PV) systems. This article presents a dataset of one-minute resolution Global Horizontal Irradiance (GHI) measurements collected at the UCSI University, Kuala Lumpur Campus, Malaysia throughout 2025. The standalone GHI measurement system was powered by a solar PV panel and battery. It utilised a Kipp & Zonen CMP3 pyranometer, an ISO 9060 spectrally flat class C thermopile pyranometer. To ensure precise data logging, the pyranometer’s analogue signals were digitalised using an ADS1115 16-bit analogue-to-digital converter (ADC) with a programmable gain amplifier. An ESP32-WROOM-32U microcontroller was used to process these signals, compute the irradiance measurements, and transmit the data to a ThingSpeak Internet of Things (IoT) server at a one-minute intervals from 07:00 to 18:59 local time. A total of 259,858 observations were retrieved out of the 262,800 expected observations. The 2942 missing observations (1.12%) were linear interpolated. Additionally, a quality control screening was conducted using Baseline Surface Radiation Network tests based on Extremely Rare Limits and Physically Possible Limits, with no observations flagged. The dataset has a combined standard uncertainty of ±4.42% and an expanded standard uncertainty of ±8.84% (k=2). This one-minute resolution GHI dataset captures rapid irradiance variations in Kuala Lumpur, Malaysia. The dataset contained daytime GHI measurements only and does not include other meteorological variables. It may support PV system modelling, simulation, and validation by enabling representation of irradiance-driven power fluctuations. The data can also be used for short-term GHI and PV power forecasting, including machine learning-based approaches. In addition, the dataset may support studies of PV power variability, PV battery energy storage system scheduling, PV output smoothing, and grid integration under high PV penetration.

Specifications Table

**Table:** 

Subject	Engineering & Materials science
Specific subject area	Renewable Energy, Sustainability and the Environment
Type of data	Processed, Analysed, Chart, Figure
Data collection	A standalone Global Horizontal Irradiance (GHI) measurement system was deployed at the UCSI University, Kuala Lumpur Campus, Malaysia. The system is powered by a solar photovoltaic panel and battery to achieve self-sustainability. GHI was measured using a Kipp & Zonen CMP3 pyranometer. The pyranometer’s analogue output was digitised by an ADS1115 16-bit analogue-to-digital converter (ADC) with a programmable gain amplifier. An ESP32-WROOM-32U microcontroller was used to read the ADC values, convert them into voltage values, and calculate the corresponding irradiance. The measurements were recorded at one-minute intervals and transmitted to a ThingSpeak server from 07:00 to 18:59 local time.
Data source location	Roof Top, Level 12, Block G, UCSI University, Kuala Lumpur Campus, Malaysia GPS Location: 3.078,842, 101.732,319.
Data accessibility	Repository name: Mendeley Data Data identification number: 10.17,632/bgch4tw79k.2 Direct URL to data: https://data.mendeley.com/datasets/bgch4tw79k/2
Related research article	None

## 1 Value of the Data

• This study presents a newly acquired Global Horizontal Irradiance (GHI) dataset for Kuala Lumpur, Malaysia, measured at a one-minute intervals from 07:00 to 18:59 daily throughout 2025. Its high temporal resolution enables the capture of short-term irradiance variability and rapid changes caused by cloud movement.
• This dataset can support solar photovoltaic (PV) system modelling, validation and simulation. Its one-minute resolution enables a more realistic representation of irradiance-driven PV power fluctuations in simulation studies.
• The dataset is valuable for short-term solar forecasting and data-driven research, including machine learning and artificial intelligence-based models for predicting GHI and PV power output.
• The data can be used as input for PV simulation models, where simulated power output may be compared with measurements from actual PV systems for model validation, as demonstrated in previous solar irradiance and meteorological data studies [1,2].
• The high-resolution GHI measurements can support power system operation, control, and grid integration studies in systems with high PV penetration. Potential applications include frequency stability analysis [3], PV power fluctuation analysis [4], PV battery energy storage system dispatch scheduling [5], PV fluctuation smoothing using BESS [6], and PV equivalent circuit modelling [7].

## 2 Background

PV system performance is highly dependent on local meteorological conditions [8]. Global Horizontal Irradiance (GHI) is one of the key factors influencing this performance. However, GHI data vary by location [9], and many commonly available meteorological datasets offer only coarse temporal resolutions [10]. When investigating PV system models for short term dynamics, extreme GHI events, and the volatility of solar irradiance, a high resolution, location specific dataset becomes necessary.

Previous studies on solar irradiance datasets have reported site specific measurements for various locations [10,11] and annual observation periods [12], with measurement intervals typically ranging from 5 min to 1 hour. Prior research [13] strongly advocates for high resolution GHI data due to its necessity in accurately modelling PV systems and ensuring their effective integration. For instance, high resolution GHI data capture rapid fluctuations in PV power output, mitigating errors in system design, energy yield predictions, and grid stability assessments, which are critical given the global surge in renewable capacity. While public datasets from ERA5, MERRA-2, and the Global Solar Atlas are useful for macro level analyses, their hourly temporal resolution cannot capture per-minute cloud transient ramps.

The need for high resolution data is particularly critical for PV system studies in Malaysia. Malaysia lies in the Northern Hemisphere and has a tropical, highly humid climate that is heavily governed by seasonal monsoons. The frequent rainstorms and fast moving clouds induce sharp fluctuations in GHI. Thus, high resolution datasets are crucial for capturing rapid shifts in irradiance.

**Fig. 1:**
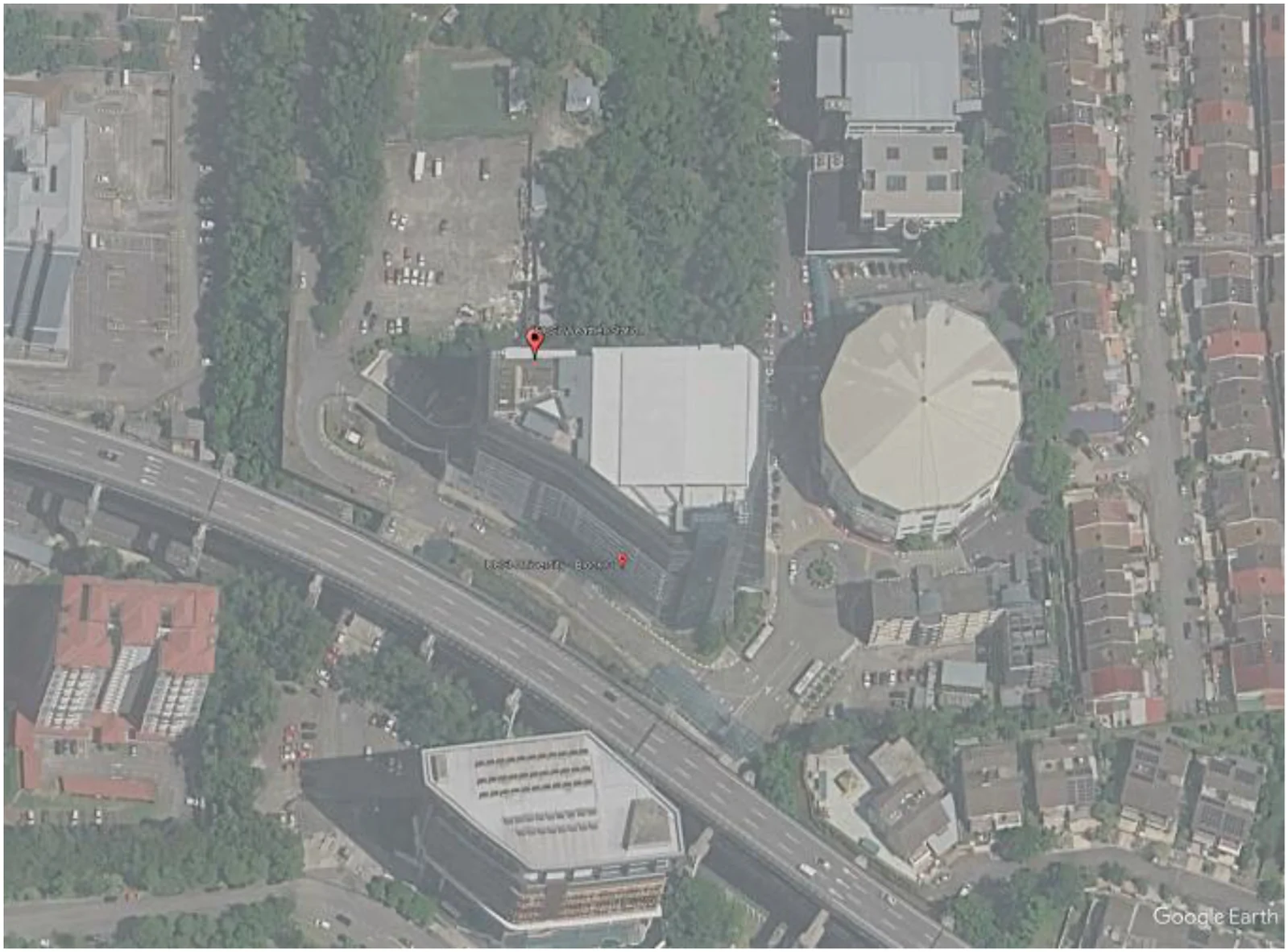
Location of the GHI measurement system (3.078842, 101.732319).

To address this gap, data with a one-minute sampling interval were recorded using the developed GHI measurement system at the UCSI University, Kuala Lumpur, Malaysia, as shown in Fig. 1. The dataset intentionally covered a predefined 12-hour daytime acquisition window from 07:00 to 18:59 local time, recorded daily throughout 2025 [14]. The final dataset consisted of a total of 262,800 data points, of which 259,858 were obtained from measurement while the remaining missing values were reconstructed using linear interpolation. Since the acquisition window was restricted to daytime hours, these measurements do not represent complete daily irradiance.

## 3 Data Description

**Fig. 2:**
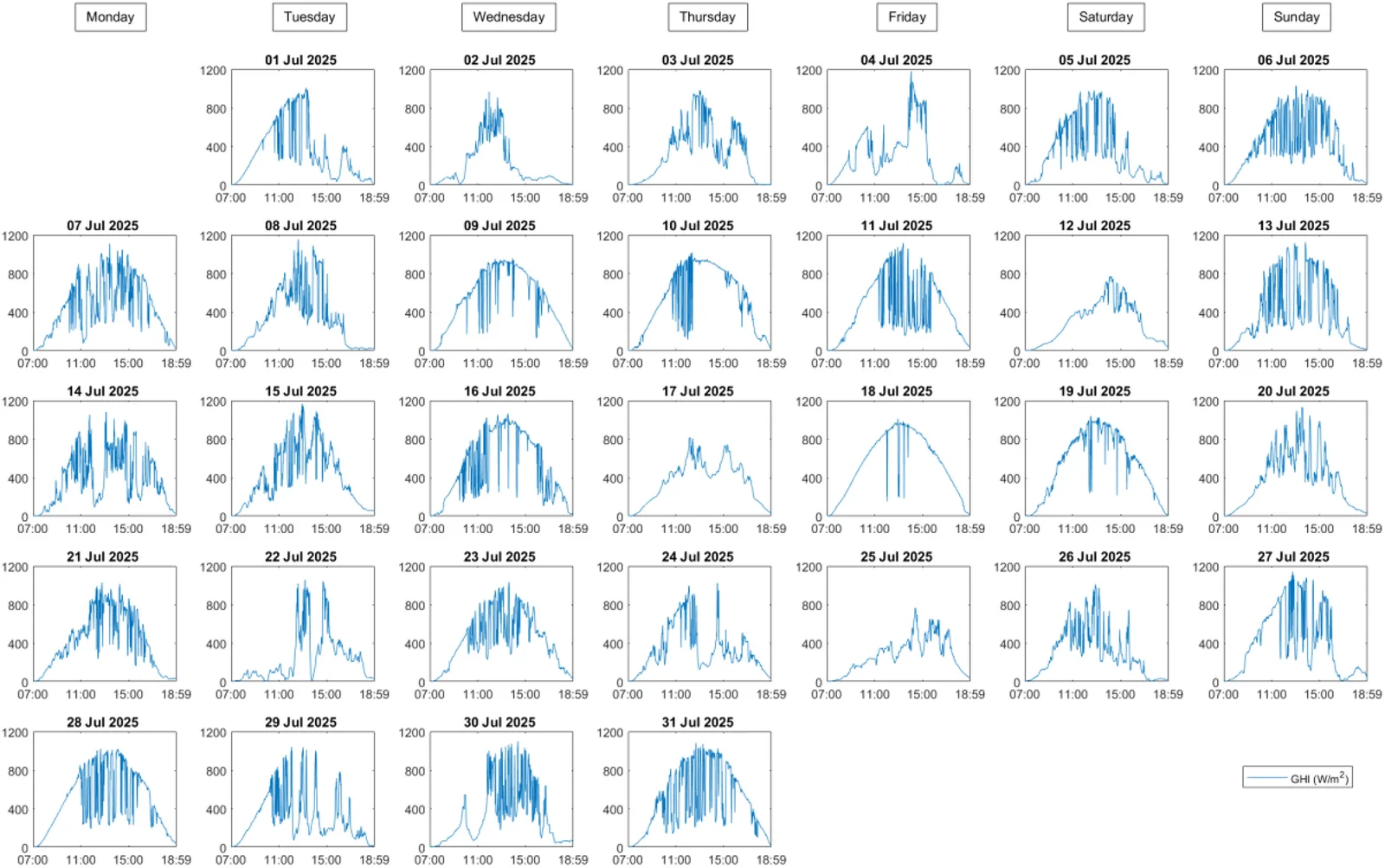
Global horizontal irradiance for July 2025.

This study presents a comprehensive dataset of GHI for Kuala Lumpur, Malaysia, captured at a one-minute resolution over the entirety of 2025 (January 1st to December 31st). This dataset is organised across 365 individuals .CSV files, with each file representing a day of GHI data recorded from 07:00 to 18:59. Fig. 2 presents the GHI data for the month of July 2025 visualised as in calendar format.

**Fig. 3:**
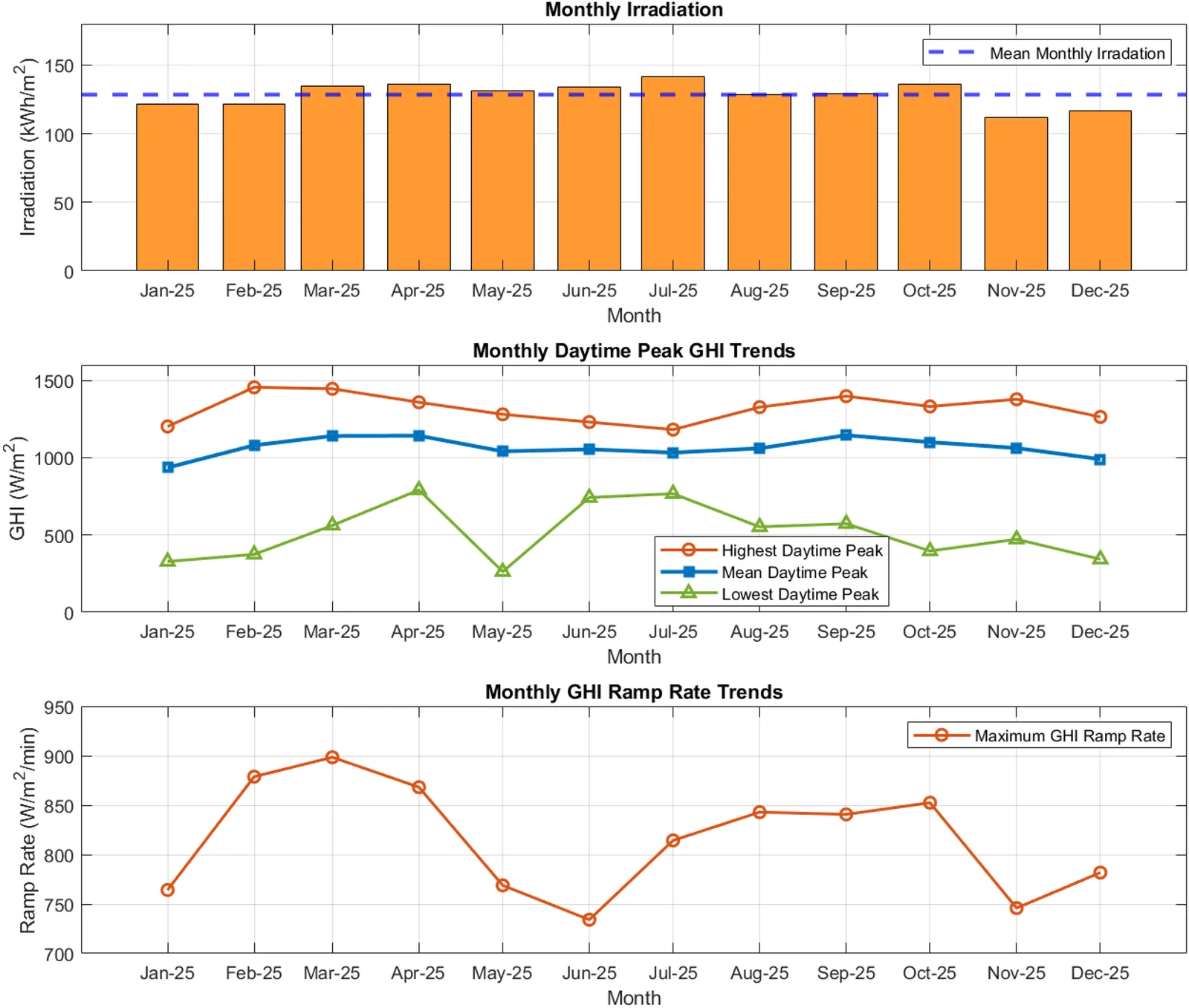
Monthly irradiation, monthly daytime peak GHI trends, monthly GHI ramp rate trends.

**Fig. 4:**
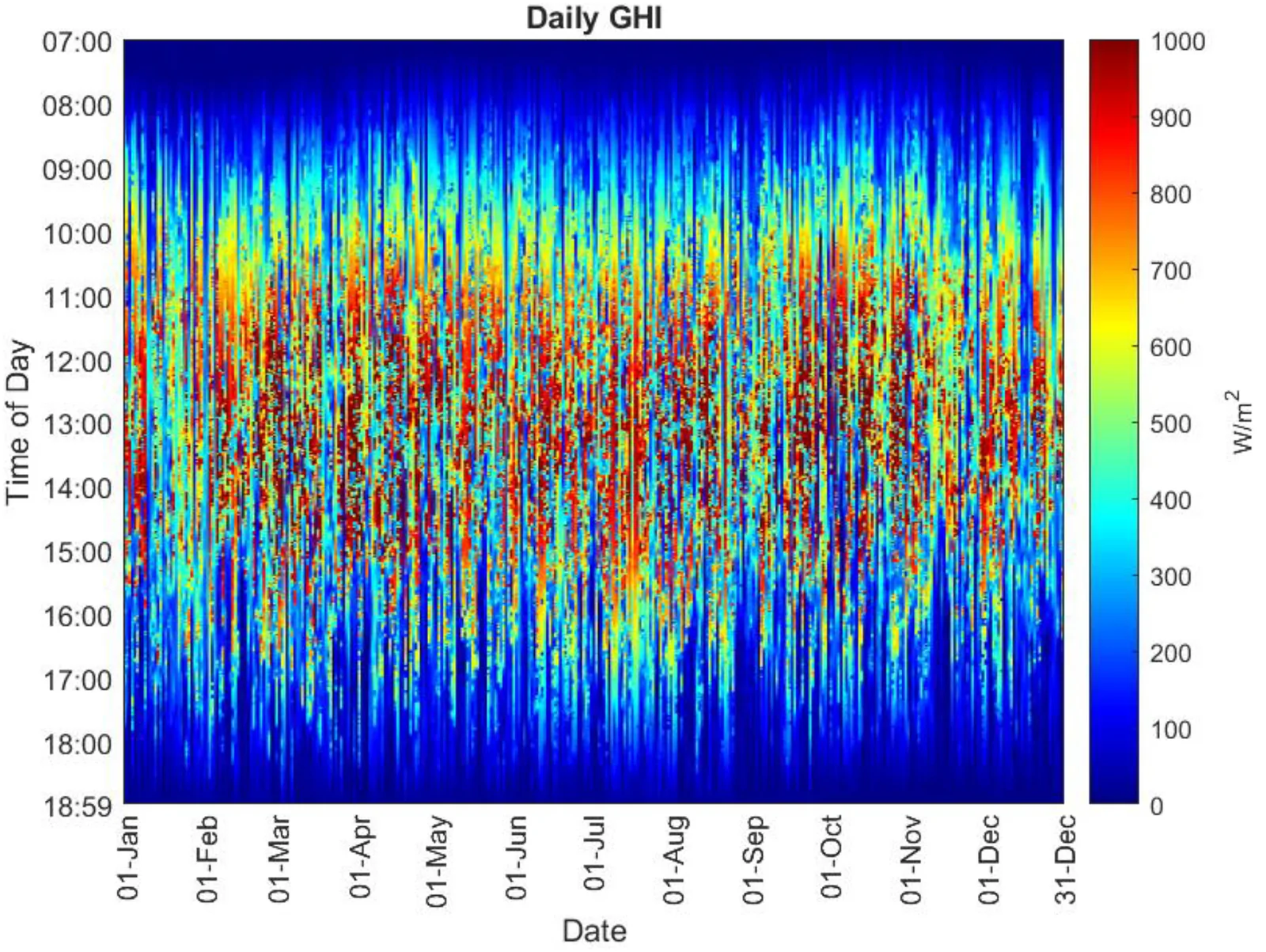
Daily GHI heatmap.

The final dataset was processed and plotted using MATLAB, as shown in Fig. 3, Fig. 4. The GHI values were summed over each month to obtain the monthly irradiation, which ranged from 112.01 kWh/m² to 141.32 kWh/m². This consistent pattern throughout the year is characterised by the absence of four distinct seasons. This behaviour is characteristic of equatorial regions, where solar elevation angles remain consistently high and day lengths are approximately 12 h year round. Across the entire year, the mean monthly irradiation was recorded at a relatively high 128.50 kWh/m², with lower irradiation observed from November to February, as well as in August.

Next, the monthly mean daytime peak GHI values were calculated by identifying the highest irradiance recorded each day and averaging these daily peak values for each month. The analysis revealed the influence of the Sun’s annual apparent path. The highest monthly mean daytime peak GHI occurred in September at 1145.65 W/m², followed closely by April at 1142.46 W/m² and March at 1140.98 W/m². These elevated values align with the periods around the March equinox (March 20th–21st) and the September equinox (September 22nd–23rd). During these periods, the Sun reaches high elevation angles over Kuala Lumpur and passes near the zenith at local solar noon, providing greater potential for solar irradiance.

Conversely, the lowest monthly mean daytime peak GHI was observed in January at 936.07 W/m², followed by December at 990.52 W/m² and July at 1033.03 W/m². These reduced values correspond to periods when the Sun is furthest south around the December solstice (December 21st –22nd) or furthest north around the June solstice (June 21st–22nd), relative to Kuala Lumpur. The lower monthly means, particularly from November to February, are further influenced by the Northeast Monsoon, which typically brings persistent rainfall and increased cloud cover, significantly attenuating solar irradiance.

While these astronomical factors define the general monthly trends, daily daytime peak GHI values are strongly influenced by local atmospheric conditions. The highest daytime peak GHI recorded was 1455.94 W/m² on February 24th. This unusually high irradiance value could be possibly due to the cloud enhancement phenomenon [15,16]. In contrast, the lowest daytime peak GHI was 262.47 W/m² on May 29th. This extremely low peak value, despite generally high solar elevation angles, is likely caused by severe atmospheric obstruction, primarily dense cloud cover and heavy rainfall during monsoon or inter-monsoon periods. Visually, the GHI data often showed substantial variation in irradiance intensity throughout the year, reflecting the influence of dynamic local weather patterns.

Further examination of the data indicates that the highest instantaneous GHI values were predominantly concentrated between 11:00 and 15:00, as shown in Fig. 4. This four-hours period corresponds to peak solar hours around local solar noon. Additionally, the daily maximum ramp rates were also analysed to characterise the dynamic behaviour of solar irradiance. The highest observed maximum ramp rate was 898.47 W/m²/min on March 18th. This rapid increase in GHI was likely associated with the sudden clearing of a significant cloud, allowing a rapid increase in available solar irradiance. This may also be attributed to relatively high solar elevation around the March equinox. Conversely, the lowest maximum ramp rate recorded was 734.14 W/m²/min on June 12th. This comparatively lower value may reflect the slightly lower solar elevation during the June solstice, together with different cloud characteristics, leading to a less pronounced increase in GHI upon cloud clearance.

In summary, this one-minute resolution dataset for Kuala Lumpur provides detailed information on the solar irradiation, solar irradiance, as well as GHI ramp rates. This information is valuable for identifying solar resource variability, understanding peak solar hours, and providing a foundational reference for comparison with other solar datasets in similar tropical environments.

## 4 Experimental Design, Materials and Methods

The data was acquired using a GHI measurement system designed to capture the variations in GHI at one-minute intervals. The system consisted of three main components: a sensor for measuring GHI, a microcontroller for data acquisition and processing and a storage system for raw data storage.

For GHI measurements, a Kipp & Zonen CMP3 pyranometer was used. It is an ISO 9060 spectrally flat class C thermopile pyranometer, recognised for its accuracy, dependability, and reliability in routine solar monitoring [17]. The pyranometer was factory calibrated via indoor side-by-side comparison under a 150 W metal-halide discharge lamp against a reference pyranometer traceable to the World Radiometric Reference via the PMO2 absolute cavity pyrheliometer at the World Radiation Centre in Davos, Switzerland. The pyranometer has a reported sensitivity constant of 13.93 μV/W/m^2^ and an expanded calibration uncertainty of ±3.4%. This uncertainty accounted for random effects, instrumental errors, directional response error and uncertainty of the transfer procedure. The expanded uncertainty corresponds to a standard uncertainty of 1.70% with a coverage factor of k=2 [18]. Its operational irradiance range is up to 2000 W/m^2^. This range is suitable for irradiance measurements in Malaysia, where peak GHI values are typically around 1000 W/m^2^.

The pyranometer produces an analogue voltage output of up to 40 mV. Therefore, an ADS1115 16-bit analogue-to-digital converter (ADC) with a programmable gain amplifier was used to measure the low-level voltage output. The ADC was connected to the microcontroller via the I^2^C digital communication protocol, enabling high resolution data acquisition while minimising noise effects. The analogue differential output voltage was digitised using the ADC, configured with a full scale input range of ±0.256 V. This configuration provides a least significant bit (LSB) resolution of 7.8125 µV. Based on the pyranometer sensitivity of 13.93 µV/W/m^2^, this corresponds to an GHI resolution of 0.561 W/m^2^. Assuming the ADC rounds the measured voltage to the nearest digital level, the maximum quantisation error is half an LSB, resulting in an ADC quantisation uncertainty of ±0.281 W/m^2^.

The ESP32-WROOM-32U microcontroller was selected because it provides integrated Wireless Fidelity (Wi-Fi) connectivity, enabling cloud-based data storage. Moreover, it supports an external antenna, offering improved communication range. The microcontroller converts the ADC digital reading to its corresponding voltage by multiplying the reading by the LSB resolution of 7.8125 µV. The calculated voltage is then divided by the pyranometer sensitivity constant of 13.93 µV/W/m^2^ to calculate the solar irradiance. The final output is expressed in Watts per square meter (W/m^2^).

**Fig. 5:**
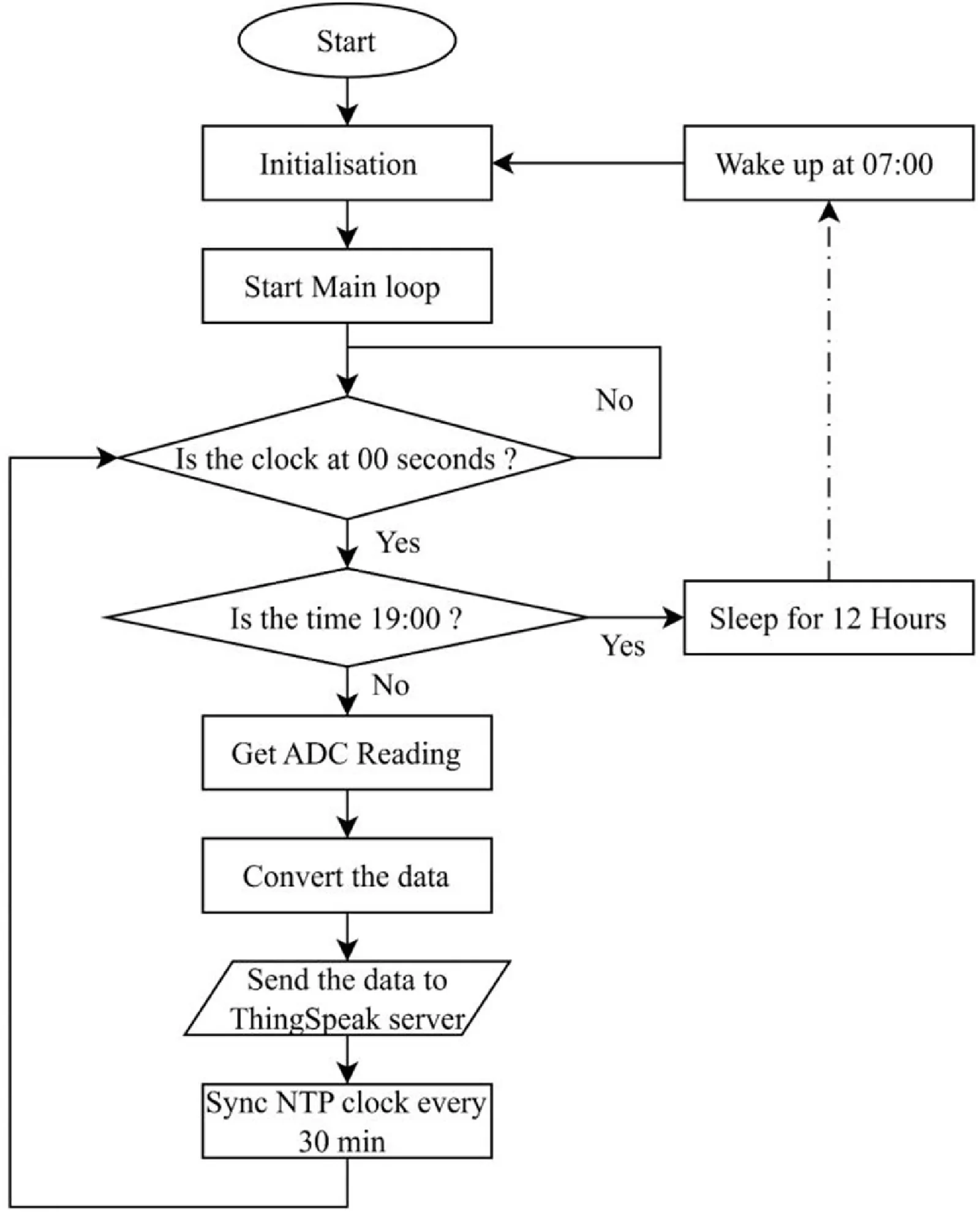
Operation logic flowchart.

The microcontroller logic flow is shown in Fig. 5. The process begins with an initialisation phase, which establishes the Wi-Fi connection, configures the ThingSpeak server Application Programming Interface (API) credentials, and sets up the necessary communication ports. During this phase, the microcontroller’s internal clock is also synchronised with a Network Time Protocol (NTP) server to ensure accurate time logging and prevent time drift throughout the data acquisition period.

After the initialisation phase, the microcontroller operates in a continuous loop that actively tracks the system time. To manage operations efficiently, the logic checks the time at the start of every minute (0 s). When the time reaches 19:00, the controller exits the active loop and enters a 12-hour sleep period, resuming normal operation at 07:00. This is designed to ensure power savings during nighttime when the solar irradiance is not available and reducing power consumption. During the operational daytime period, the system continues to acquire the irradiance readings.

The data are then transmitted to the ThingSpeak server. The free tier of ThingSpeak permits up to three million messages annually. As the microcontroller transmits 720 data points during each 12-hour daytime period, the system is expected to transmit 262,800 data points per year, which is well within the platform’s limits. Upon receipt of the data, ThingSpeak automatically timestamps them. Although network latency introduces a delay of a few seconds between data acquisition and server reception, this discrepancy is negligible and does not compromise the minute level accuracy of the timestamps. The final step in the execution loop ensures that the microcontroller’s internal clock remains accurate. To prevent time drift, the system re-synchronises with the NTP server every 30 min.

**Fig. 6:**
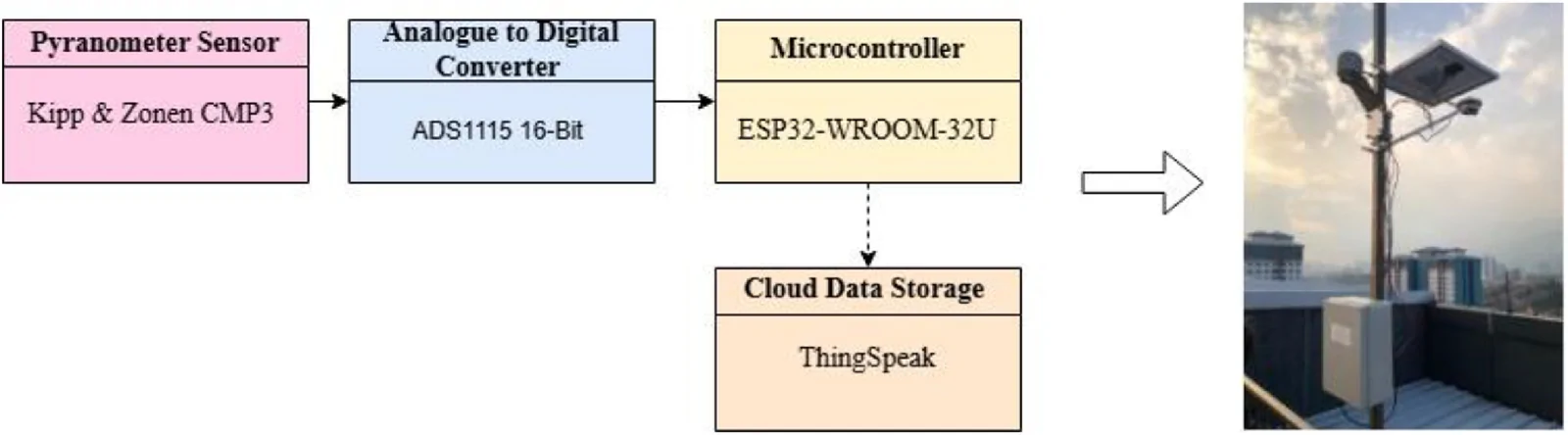
GHI measurement system overview.

The complete system setup and its components are shown in Fig. 6. The analogue signal generated by the pyranometer is fed into the ADC it is digitised and read by the microcontroller. The microcontroller then calculates the corresponding irradiance values and transmits the data to cloud storage.

**Fig. 7:**
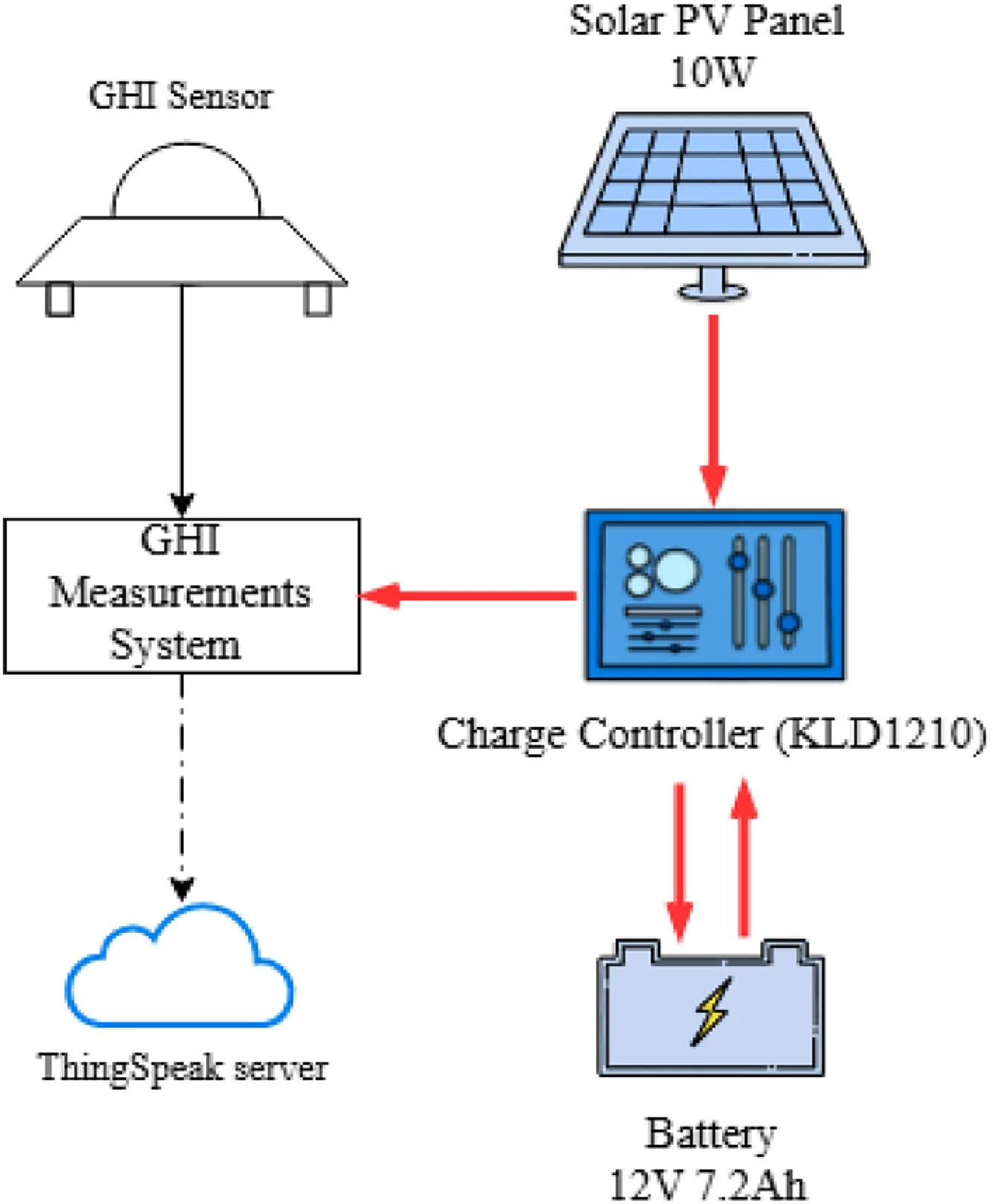
Power supply system overview.

In additional, a solar PV and battery power system was used to provide constant and uninterrupted power supply for the microcontroller. The system consists of a 10 W solar panel and a 12 V, 7.2 Ah battery. The battery and the solar panel were connected through a solar charge controller, KLD1210. The setup of the power system was designed to allow the GHI measurement system to operate continuously for >24 h, ensuring uninterrupted data acquisition. An overview of the power supply system is shown in Fig. 7.

The GHI measurement system was installed on the rooftop of Block G, a 12-level academic block at the UCSI University Kuala Lumpur campus (3.078842, 101.732319). The site has a ground elevation of approximately 80 m, with the building adding 45 m in height, resulting in a total elevation of approximately 135 m. The installation location was carefully selected to ensure an unobstructed view of the Sun’s path throughout the year. The system was mounted on a vertical pole positioned near the edge of the rooftop. The pyranometer was mounted on an extension arm, which was oriented northwest at an azimuth of 337° clockwise from true north relative to the pole.

After daily extraction of GHI data from the ThingSpeak server, a data completeness check was conducted to identify missing observations. A total of 262,800 observations was expected for the entire acquisition period. However, only 259,858 observations were successfully retrieved, resulting in 2942 missing observations (1.12%). The missing observations were due to occasional discontinuities caused by server latency and Wi-Fi connectivity issues. These missing timestamps were inserted to maintain a continuous and regularly sampled time series and the corresponding GHI values were estimated using linear interpolation [13].

The highest number of missing observations occurred on January 20th, with 43 non-consecutive missing observations. Across the entire dataset, the longest consecutive missing data gap occurred on June 20th and lasted 37 min. The distribution of consecutive missing data gaps is presented in Table 1. Among the 1713 missing data gaps, 1456 lasted 1 min while 235 lasted 2–10 min, accounting for 52.86% and 33.45% of all missing data, respectively. Meanwhile, gaps longer than 10 min were infrequent, accounting for 13.70% of all missing observations, equivalent to only 0.153% of the 262,800 expected observations. As most missing data occurred in short duration gaps, linear interpolation was considered suitable for estimating missing observation and maintaining a continuous one-minute time series.

**Table 1:** Gap duration distribution.

Gap Duration (min)	Number of Missing Data Gaps	Total Missing Observation	Share of Missing Data (%)	Share of Expected Observations (%)
1	1456	1555	52.855	0.592
2 - 5	198	635	21.584	0.242
6 - 10	37	349	11.863	0.133
11 - 20	17	265	9.007	0.101
21 - 30	3	66	2.243	0.025
31 - 40	2	72	2.447	0.027
Total	1713	2942	100.000	1.119

Next, a quality control screening was applied to the annual dataset by utilising the Baseline Surface Radiation Network (BSRN) test, as detailed in [19]. This test incorporates Extremely Rare Limits (ERL) and Physically Possible Limits (PPL), which serve as upper bounds to assess data quality. ERL values represent measurements that are physically plausible but statistically exceptional, indicating a need for further verification. Meanwhile, PPL values signify measurements that cannot occur under real atmospheric conditions, indicating a potential measurement error. Both ERL and PPL flags identify data that deviate significantly from expected values. The extraterrestrial normal irradiance (ETN) and solar zenith angle (SZA) values were computed based on the location of 3.078842, 101.732319 and Malaysia’s UTC+8 time zone. Following the application of this quality control screening, the dataset successfully passed the screening, with no values being flagged under either the ERL or PPL criteria [17].

Moreover, the uncertainties of the pyranometer, according to the CMP3 datasheet [17], were tabulated in Table 2 to establish the uncertainty budget and calculate the combined standard uncertainty. According to the manufacturer, the pyranometer sensitivity remains stable while stored in its original packaging prior to initial deployment. Therefore, a non-stability uncertainty of ±1.0% was assumed for the 1-year outdoor operational period, based on the manufacturer-specified non-stability of <1% per year. A typical field soiling and dust uncertainty of 2.5% was assumed. The combined standard uncertainty was calculated using root-sum-square method, resulting in ±4.42%. The expanded standard uncertainty was ±8.84%, with a coverage factor of k=2 [18]. To minimise measurement errors during the 1-year data acquisition period, routine maintenance was performed, including periodic cleaning of the optical glass dome to prevent excessive soiling accumulation and verification of horizontal alignment using the integrated bubble level.

**Table 2:** Uncertainty budget.

Source of Uncertainty	Bound (%)	Distribution	Standard Uncertainty (%)
Expanded calibration uncertainty	±3.400	Normal	±1.700
Temperature response	±4.000	Rectangular	±2.309
Non-linearity	±3.000	Rectangular	±1.732
Directional response^1^	±2.000	Rectangular	±1.155
Zero offsets (thermal radiation)^1^	±1.500	Rectangular	±0.866
Zero offsets (temperature change)^1^	±0.500	Rectangular	±0.289
Tilt response	±1.500	Rectangular	±0.867
Non-stability	±1.000	Rectangular	±0.577
Spectral selectivity	±3.000	Rectangular	±1.732
ADC quantisation^1^	±0.028	Rectangular	±0.016
Field soiling & dust	±2.500	Rectangular	±1.443
Combined standard uncertainty	-	-	±4.422

1 The uncertainty bound is normalised at 1000 W/m^2^.

## Limitations

The data collected using the designed GHI measurement system have several limitations. Since solar irradiance varies inherently across geographical locations, the data presented are representative only of the specific site where the measurement system was installed and do not necessarily reflect the GHI conditions across Malaysia. Moreover, the data reflects the site-specific environmental conditions and may be affected by local weather conditions and topography. Furthermore, the dataset includes daytime measurements only and does not include other metrological parameters, such as wind speed, humidity, and direct normal irradiance. These limitations may restrict its applicability in certain advanced PV performance analyses. The dataset is subjected to a combined standard uncertainty of ±4.42% and an expanded standard uncertainty of ±8.84% (k=2).

## Ethics Statement

The authors declare that the current work does not involve human subjects, animal experiments, or any data collected from social media platforms.

## CRediT authorship contribution statement

**Chun Lim Siow:** Methodology, Formal analysis, Funding acquisition, Writing – original draft. **Chee Wern Hew:** Methodology, Formal analysis, Investigation, Software, Data curation, Visualization, Writing – original draft. **Ousama M.T. Ajami:** Validation, Data curation, Investigation, Writing – review & editing. **Rodney H.G. Tan:** Conceptualization, Resources, Supervision, Validation, Project administration, Writing – review & editing.
